# Increased Risk of Stroke in Patients of Concussion: A Nationwide Cohort Study

**DOI:** 10.3390/ijerph14030230

**Published:** 2017-02-25

**Authors:** Shih-Wei Liu, Liang-Chung Huang, Wu-Fu Chung, Hsuan-Kan Chang, Jau-Ching Wu, Li-Fu Chen, Yu-Chun Chen, Wen-Cheng Huang, Henrich Cheng, Su-Shun Lo

**Affiliations:** 1 Department of Emergency Medicine, National Yang-Ming University Hospital, I-Lan 260, Taiwan; shihweiliu123@gmail.com (S.-W.L.); horus7855@yahoo.com.tw (L.-C.H.); wolfchung2001@yahoo.com.tw (W.-F.C.); 2 Department of Neurosurgery, Neurological Institute, Taipei Veterans General Hospital, Taipei 112, Taiwan; hsuankanchang@gmail.com (H.-K.C.); wchuang@vghtpe.gov.tw (W.-C.H.); hc_cheng@vghtpe.gov.tw (H.C.); 3 School of Medicine, National Yang-Ming University, Taipei 112, Taiwan; hc_cheng@vghtpe.gov.tw (Y.-C.C.); sslo@ymuh.ym.edu.tw (S.-S.L.); 4 Department of Medical Research and Education, National Yang-Ming University Hospital, I-Lan 260, Taiwan; 5 Institute of Hospital and Health Care Administration, National Yang-Ming University, Taipei 112, Taiwan; 6 Institute of Pharmacology, National Yang-Ming University, Taipei 112, Taiwan

**Keywords:** concussion, hemorrhagic stroke, ischemic stroke, cohort, and traumatic brain injury (TBI)

## Abstract

Long-term morbidities can develop after traumatic brain injury (TBI). Some studies have suggested that the risk of stroke is higher after TBI, but the association between concussion and stroke remains unclear. Using a national cohort, the authors analyzed the incidence of both hemorrhagic and ischemic strokes in patients with previous concussion. A representative cohort of approximately one million people was followed up for four years. Patients with new-onset concussion were identified (*n* = 13,652) as the concussion group. Subsequently, the incidence rates of later stroke events in the concussion group were compared to a sex-, age- and propensity score–matched comparison group (*n* = 13,652). The overall incidence rate of stroke in the concussion group was higher than that of the comparison group (9.63 versus 6.52 per 1000 person-years, *p* < 0.001). Significantly higher stroke risk was observed in the concussion group than in the comparison group (crude hazard ratio 1.48, *p* < 0.001; adjusted HR 1.65, *p* < 0.001). In the concussion group, the cumulative incidence rates of both ischemic stroke and hemorrhagic stroke were higher than those of the comparison group (8.9% vs. 5.8% and 2.7% vs. 1.6%, respectively, both *p* < 0.001). Concussion is an independent risk factor for both ischemic and hemorrhagic strokes. Prevention and monitoring strategies of stroke are therefore suggested for patients who have experienced concussion.

## 1. Introduction

Traumatic brain injury (TBI) is a common medical problem in the United States with an estimated incidence of approximately 1.5 million injuries occurring each year [1]. Of these TBIs, approximately 85% are considered mild (i.e., concussion). The common causes of concussion treated in emergency departments are falls, motor vehicle collisions, unintentional head trauma, assaults, and sports [2].

Studies have demonstrated that many post-traumatic morbidities can develop after the initial brain insult. These consequences included a decline in cognition function, dementia, chronic traumatic encephalopathy, Parkinson’s disease, epilepsy, and psychiatric and metabolic dysfunctions [3,4,5,6,7,8,9,10,11]. Two recent studies raised the concern about TBI and stroke [12,13]. A retrospective cohort study in California demonstrated that in the trauma patients visiting the emergency department, TBI was independently associated with subsequent ischemic stroke, regardless of the severity or subtype of TBI [12]. Another cohort study demonstrated that TBI was associated with 10.21, 4.61, and 2.32 times greater risk of stroke three months, one year and five years after the injury, respectively [13]. However, these two studies shared the common caveat that all TBI patients with various types of injury were included. These types included mild head injuries (e.g., concussion), which required only observation, to severe ones (e.g., subdural hemorrhage, brain contusion, and diffuse axonal injury), which required surgery. The result of increased stroke risk was thus derived from a mixture of different kinds of TBI patients. However, it is reasonable to infer that the long-term consequences of mild TBIs (i.e., concussion) should be very different from severe TBIs. However, the actual risk of subsequent stroke in patients with mild TBI remains elusive.

Unlike severe TBIs which frequently involve contusion, edema and hemorrhage of the brain tissue, concussion has little structural damage and is considered the mildest type of TBI. Although brain concussion is usually not a life-threatening condition, there is a growing concern about this mild TBI, as it increasingly could be sports-related in modern society [14,15]. Although concussion often recovers spontaneously after a period of time, its long-term effects remain uncertain. Furthermore, it is difficult to evaluate the subsequent medical conditions of these concussion patients because they are usually not followed up for long. The authors therefore designed this population-based cohort study to investigate the association between concussion and stroke.

This study aimed to clarify the association between concussion and stroke using a comprehensive national health insurance database. In Taiwan, the monopolistic health system sponsored by the government covers the entire population. Therefore, using the database, this cohort study had a uniquely high (almost complete) rate of follow-up. The authors took advantage of this to compare the incidence rates of stroke in patients with brain concussions to a deliberately matched control cohort. To date, this is the first report to specifically address such an issue. 

## 2. Materials and Methods 

### 2.1. Data Source and Ethic Concern

The National Health Insurance (NHI) program in Taiwan has provided unrestricted access to health care services for the entire population of 23 million people since 1995. This government-sponsored health insurance system monopolistically covers up to 99% of the population in Taiwan, and has contracts with over 97% of all the medical service providers. The National Health Insurance Research Database (NHIRD), provided by NHI, contains comprehensive information on insured subjects, including gender, date of birth, residential or work area, dates of clinical visits, the International Classification of Diseases (Ninth Revision) Clinical Modification (ICD-9-CM) codes of diagnoses, details of prescriptions, expenditure amounts and outcome at hospital discharge (recovered, died, or transferred out).

This study was initiated after approval from the Institutional Review Board of Taipei Veteran’s General Hospital, Taiwan (Project Identification Code: 2012-10-008BC). Since all identifying personal information in the NHIRD was stripped before analysis, the Institutional Review Board waived the requirement for written informed consent from each of the patients involved. 

### 2.2. Study Cohort and Study Population

This is a population-based retrospective cohort study. A random representative sample with approximately one million people (*n* = 999,997) was enrolled from 1 January 1998 to 31 December 2005.

The concussion group consisted of patients who had a first episode of concussion and were aged >40 years. Those who had the diagnosis code of concussion 850.X according to the International Classification of Disease, 9th Version (ICD-9) at either an out-patient clinic or emergency department were included. The first episode of concussion was defined by: (1) No other intracranial injury (ICD-9 801.x–805.x, 851.x–854.x, 907.0) diagnosed one week before and after the diagnosis of concussion; (2) First concussion event happened between 1 January 1998 and 31 December 2005 without any intracranial injury identified at least one year before. 

A comparison group consisted of age-, sex- and propensity score–matched persons without concussion randomly extracted from this representative cohort database at a case-comparison ratio of 1:1. The propensity scores were calculated by age, sex, hypertension, diabetes, arrhythmia, cardio-vascular diseases, and use of aspirin, lipid-lowering drugs, nitrates, and non-steroidal anti-inflammatory drugs (NSAIDs). An index date was designated to match each case’s index date (first diagnosis date for the concussion patients).

**Figure 1:**
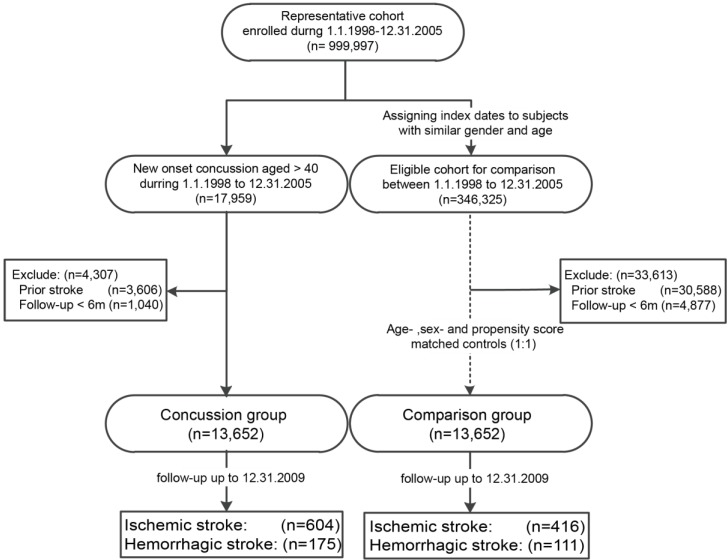
Flow of data processing.

Patients in both groups with prior stroke events or an inadequate follow-up period (<6 months) were excluded. Patients of both groups (*n* = 13,652 each) were tracked for four years until 31 December 2009. The flow of data processing is summarized in Figure 1. 

### 2.3. Covariates and Study Endpoint

Co-morbidities, which included hypertension (ICD-9 code, 401.x–405.x), diabetes mellitus (250.x), arrhythmia (426.x–427.x), and coronary heart disease (410.x–414.x), were designated as covariates. Exposure to medications, including aspirin, anti-coagulants, lipid lowering drugs, and nitrates was also included as a covariate in the analysis. These were determined by the presence of either diagnostic codes in the outpatient records or discharge codes in the medical chart, six months before the index dates to the date of the outcome event or the end of follow-up. To further avoid selection bias in matching, the propensity score method was applied and adjustments of baseline differences between the groups were calculated by logistic regression analysis for all covariates [16]. Since the variables were known risk factors of stroke, by matching the two groups with propensity score, the selection bias as well as confounders thus could be reduced.

The study endpoint was the event of stroke-related hospitalization determined by the hospitalization records with a discharge diagnostic code of stroke (ICD-9 code, 430.x–435.x) or death by the date of 31 December 2009. Hemorrhagic strokes (430.x–432.x) versus ischemic strokes (433.x–435.x) were also stratified for analysis. 

### 2.4. Statistical Analysis

All of the data were linked using the SQL server 2008 (Microsoft Corp., Redmond, WA, USA) and analyzed by SPSS software (SPSS, Inc., Chicago, IL, USA). Chi-square and independent *t*-tests were used to assess differences in age, gender and co-morbidities between the concussion and comparison groups. After matching with age, sex and propensity scores, the Kaplan-Meier method and Log-rank test were used to estimate and compare the incidence rates of hospitalizations for stroke. The Cox proportional hazard model was used to compare the incidence rates of stroke (hemorrhagic and ischemic) between the two groups after adjustment for the aforementioned covariates. A two-tailed level of 0.05 was considered statistically significant. 

### 2.5. Quantitative Bias Analysis

Probabilistic sensitivity analysis was conducted using R 3.2.2 (The R Foundation for Statistical Computing, Vienna, Austria) to evaluate the influence of exposure misclassification (validity of concussion) [17]. The misclassification bias corrected risk ratio was calculated under the assumption of sensitivity of visit for concussion follows a uniform distribution ranging from 80% to 100% (i.e., 80% of patients will visit a doctor) and the specificity of concussion visit follows a uniform distribution ranging from 50% to 100% (i.e., 50% of visits were over-diagnosed as concussion). The median smoking-adjusted stroke incidence rate ratio was calculated under the assumption of a prevalence ranging from 15% to 18% and risk ratio for any stroke episode for smokers ranging from 1.61 to 9.06 [18]. Both bias corrected and smoking-adjusted incidence rate ratio were compared to observed incidence rate ratios to quantify the effect of bias.

## 3. Results

A total of 27,304 subjects (i.e., 13,652 concussion patients, and 13,652 age-, sex- and propensity score–matched comparisons) were followed up for 154,657.3 person-years. The mean age was 56.26 ± 0.07 years at the index dates of the first concussion events, and 46% of the subjects were male. The overall incidence rate of stroke was 8.07 per 1000 person-years.

All the characteristics and comorbidities between the concussion and the comparison groups, including age at index date, sex, hypertension, diabetes, arrhythmia, coronary artery disease, medication exposure, were very similar. The distribution of demographic characteristics, selected comorbidities, and exposure to medication are shown in Table 1.

### 3.1. Incidence of All Strokes

At the end of follow-up, a total of 779 patients had a stroke in the concussion group (i.e., 604 ischemic stroke plus 175 hemorrhagic stroke). On the other hand, a total of 527 patients had a stroke in the comparison group (i.e., 416 ischemic stroke plus 111 hemorrhagic stroke) (Figure 1).

The overall incidence rate of stroke was 8.07 per 1000 person-years in the entire sample. The incidence of stroke was higher in the concussion group (9.63 per 1000 person-years) than in the comparison group (6.52 per 1000 person-years, *p* < 0.001). Patients in the concussion group were more likely to develop stroke than the comparison group. The crude hazard ratio (HR) of stroke was 1.48 (95% confidence interval (CI), 1.32–1.66, *p* < 0.001) for the subjects with a pervious concussion. After adjustment for covariates, the adjusted HR was 1.65 (95% confidence interval (CI), 1.47–1.85, *p* < 0.001) (Table 2).

**Figure 2:**
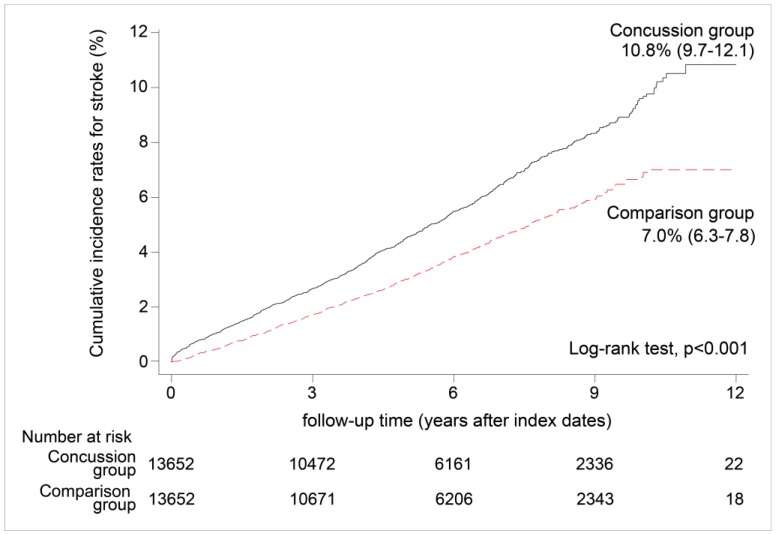
Cumulative incidence rates for stroke in the concussion group and comparison group (*n* = 27,304, 1998–2005).

The cumulative incidence rate of stroke for the concussion group was 10.8% (95% CI, 9.7%–12.1%), which was significantly higher than that of the comparison group (7.0%; 95% CI, 6.3%–7.8%, log-rank test, *p* < 0.001) (Figure 2). Subjects in the concussion group were more likely to develop stroke than those in the comparison group.

### 3.2. Hemorrhagic Stroke vs. Ischemic Stroke

A subgroup analysis of the two types of stroke (i.e., hemorrhagic and ischemic) in the cohort was subsequently conducted. Patients of the concussion group were more likely to develop stroke, either the hemorrhagic or ischemic type, than the those of the comparison group. Using the comparison group as a reference, the crude hazard ratios of hemorrhagic and ischemic stroke in the concussion group were 1.58 (95% CI, 1.24–2.02, *p* < 0.001) and 1.46 (95% CI, 1.29–1.66, *p* < 0.001), respectively. After adjusting for covariates, the adjusted hazard ratios of hemorrhagic and ischemic stroke were, respectively, 1.73 (95% CI, 1.36–2.20, *p* = 0.002) and 1.62 (95% CI, 1.43–1.84, *p* < 0.001) for patients of the concussion group (Table 3).

**Figure 3:**
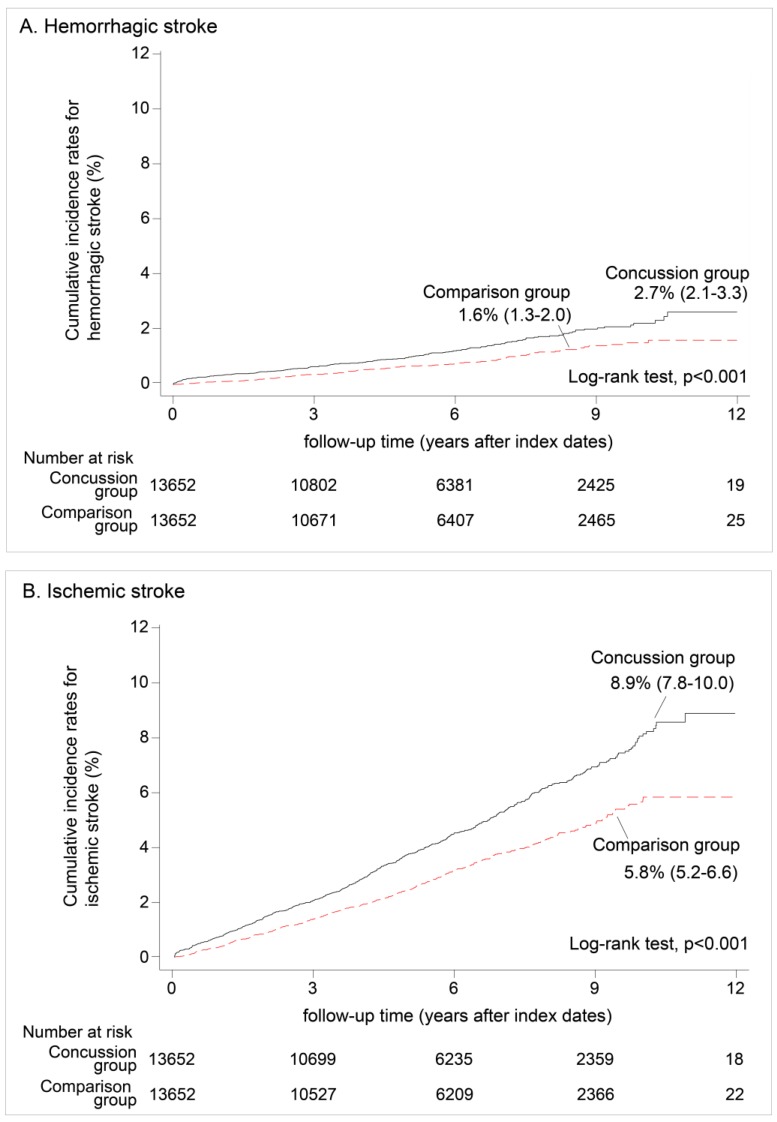
Cumulative incidence rates of (**A**) hemorrhagic and (**B**) ischemic strokes of the concussion group and the comparison group (*n* = 27,304, 1998–2005).

The cumulative incidence rates of both hemorrhagic and ischemic stroke of the concussion group were significantly higher than those of the comparison group. The cumulative incidence rates of hemorrhagic and ischemic stroke of the concussion group were 2.7% (95% CI, 2.1%–3.3%) and 8.9% (95% CI, 7.8%–10.0%), respectively. These aforementioned incidence rates were significantly higher than those of the comparison group, 1.6% (95% CI, 1.3%–2.0%) and 5.8% (95% CI, 5.2%–6.6%) (log-rank tests, both *p* < 0.001) (Figure 3).

### 3.3. Probabilistic Sensitivity Analysis

The influence of misclassification bias and immeasurable confounders (smoking status) is minimal for the current result. The misclassification bias–corrected risk ratio is 1.86 with 95% simulation limits of 1.46 and 10.86. The median smoking-adjusted stroke incidence rate ratio is 1.77 with 95% simulation limits of 1.45 and 2.55. Both rate ratios were similar to the observed incidence rate ratio (IRR = 1.48, 95% CI = 1.32–1.66) (Table 2) and suggested a minimal effect on the current result.

## 4. Discussion

The authors conducted a national cohort study to investigate patients with brain concussion and the risk of subsequent stroke. They compared 13,652 patients who had concussions to 13,652 matched control patients of similar age, sex and medical comorbidities over four years of simultaneous follow-up. The investigation demonstrated that patients of the concussion group were more likely to suffer from stroke than the comparison group (crude HR = 1.48, *p* < 0.001; adjusted HR 1.65, *p* < 0.001). When looking into the specific types of stroke (hemorrhagic and ischemic), patients who had had a previous concussion also had significantly higher risks of stroke than those of the comparison group (adjusted HR = 1.73 and 1.62, *p* = 0.002 and *p* < 0.001, respectively). 

The study had the merit of comprehensive coverage of the entire nation’s population because of the government-operated monopolistic health insurance system. The nearly perfect follow-up of these concussion patients yielded the unique opportunity to identify every subsequent stroke event. The head-to-head comparison with a group of well-matched healthy individuals of very similar demographics made the control optimal. Therefore, this study provided evidence that patients who have experienced concussion should be cautioned about subsequent stroke. Although this is a retrospective cohort, it allowed research that could not be conducted in the fashion of prospective randomization.

The sequelae of concussion, a mild TBI, might be underestimated [19,20]. The World Health Organization Collaborating Center for Neurotrauma Task Force has estimated that up to 70%–90% of all traumatic brain injuries are treated as a mild TBI or concussion [21]. Some researchers have suggested that nearly 3.8 million sports-related traumatic brain injuries occur in the United States each year and this number may underestimate the actual injuries [22]. Furthermore, sports-related mild TBIs or concussions in the US cost the health care system about $56 billion every year [23]. The impact of stroke on health care systems even exceeds that of mild TBIs. Stroke is now the second most common cause of death worldwide, and a major global cause of disability [24]. Stroke is undoubtedly a major issue in national health care systems and socio-economies. Identification and prevention of risk factors has always been an important task. The present study identified the association between both diseases and should not be overlooked.

Two papers have discussed the relationship between TBIs and stroke. Chen et al. found a higher risk of stroke within all types of TBI patients without analysis of TBI subgroups and severity [13]. Burke et al. focused on trauma patients at an emergency department and discovered that subsequent ischemic stroke was more likely to occur after head trauma than all other kinds of trauma. Their analysis also showed an increased risk for ischemic stroke regardless of TBI subtypes, including concussion [12]. Other studies following these two have further exposed the association between stroke patients and previous TBI [25]. One study focused on the relationship between ischemic stroke and mild TBI and revealed mild TBI as a strong risk factor for ischemic stroke [26]. 

Our nationwide, population-based study showed that concussion, also known as mild TBI, was an independent risk factor associated with both hemorrhagic and ischemic stroke. Since the patients and physicians dealing with concussions often neglected or were unaware of this long-term complication, the results of the current study provide important information to health care providers and are compatible with the above-mentioned reports. Furthermore, we demonstrated that even concussion, the most minor form of head injury, can result in major long-term neurologic sequelae, including ischemic and hemorrhagic stroke. 

Data in the literature addressing the pathophysiology and mechanism of stroke after concussion are scarce. Although some studies imply that cerebrovascular response after concussion could play a role [27], future studies are required to clarify the mechanism of the elevated risk of stroke after concussion. As cerebral autoregulation has the intrinsic ability to maintain stable cerebral blood flow (CBF), CBF could decrease immediately following both severe and mild TBI and could remain low for extended periods of time, depending on the severity of injury. In pediatric studies, CBF increased after mild TBI on the day of injury and then subsequently decreased in the following days. A similar presentation was observed in older patients following mild TBI [28]. The autoregulatory mechanism was sensitive to any trauma or damage, regardless of it being mild or severe TBI [29,30]. The lowering of cerebral perfusion pressure (CPP) caused by increased intracranial pressure, brain swelling, also is thought to impair autoregulation. Therefore, the impaired autoregulation would cause the brain to be more vulnerable to inadequate CPP, which could result in ischemic insults. Moreover, if systemic arterial hypotension ensued, which is not uncommon in major trauma, it could further devastate the autoregulation and cause a further decrease in CBF [30]. The vicious cascade might end up with rapid deterioration of neurological function as well as organic damage to the neuronal tissue. 

Coagulopathy after TBI might also be involved partially in the mechanism of subsequent neurological sequelae. The loss of equilibrium among the physiologically regulated coagulation factors could lead to either hyper- or hypo-coagulable states, including micro-thrombosis, secondary ischemia, and progression of hemorrhagic lesions [31]. A link between the severity of ischemia and the density of intravascular micro-thrombosis in brain tissue of TBI patients was reported [32]. After TBI, the micro-thrombosis sometimes could continue to develop in a delayed manner [33]. A similar condition was found in an animal model of TBI [34]. A drastic phenomenon demonstrated in the microcirculation of the traumatic cerebral penumbra was the formation of micro-thrombosis in up to 77% of all investigated venules, and in up to 40% of the arterioles. The micro-thrombosis was present as soon as 30 min after trauma, and the maximum level of thrombus formation was seen between 60 and 90 min after injury. Not all of these thrombi would occlude the affected vessel immediately. However, as time passed, the micro-thrombosis tended to develop more, and could completely occlude venules and arterioles [34], which would result in delayed cerebral ischemia.

From clinical data presented in the literature, ischemic stroke preceded by mild head injury has been described in pediatric patients [35,36,37,38]. Most of the patients reported in the literature had ischemic stroke after a head injury within several hours or days without the presence of artery dissection. However, one pediatric cohort study identified mild head trauma as a six-times-higher risk factor for basal ganglion and internal capsule stroke [39]. The study proposed that artery spasm induced by trauma, the mechanical disruption of the flow in the perforating branch, or intimal trauma with subsequent thrombosis may explain the mechanism of stroke. Data for adults were less commonly reported. 

There are some limitations to our study. The data source from NHI does not include detailed clinical information, such as the severity of trauma, radiological images, or the exact mechanism of concussion (i.e., motor vehicle accidents, falls, sport-related injury, or violence), which limited additional subgroup analysis. Several risk factors of stroke, including body mass index, diet pattern, physical activity level, cigarette smoking, alcohol consumption, and the severity of risk factors were not available for analysis in the present data. Nevertheless, the current study has taken the major risk factors of stroke into account, which included age, sex, chronic comorbidities, and medication exposure. These factors were adjusted for control, and the authors used propensity score matching between the two groups, which ameliorated a substantial portion of the bias. Thus, the demographic characteristics of both groups were similar. Furthermore, patients with any prior stroke diagnosis were excluded in our cohort study, which minimized the chance of reverse causation (i.e., patients with previous stroke might be paraplegic or have neurologic deficits that caused unstable gait that could increase the risk of fall and head injury). Finally, confirmation of the diagnosis of stroke in the current study may be questioned. However, a published study of strokes recorded in the NHIRD, confirmed by means of a review of each patient’s actual radiological images and medical records, confirms that the accuracy of diagnoses was as high as 97.85% [40]. The report concluded that the NHIRD is a valid resource for population research on strokes. Therefore, the calculation of stroke events after TBI in the current study is likely reflecting the reality. Further investigations over a longer time and of a larger number of patients may further corroborate the risk of cerebrovascular diseases after head injury. 

## 5. Conclusions 

Concussion is an independent risk factor for both ischemic and hemorrhagic strokes. Prevention and monitoring strategies of stroke are therefore suggested for patients of concussion. 

## Figures and Tables

**Table 1:** Baseline characteristics of comparison group and concussion group (*n* = 27,304, 1998–2005).

Baseline Characteristics			Comparison Group		Concussion Group		*p*-Value
			*n* = 13,652	(%)	*n* = 13,652	(%)	
Age at index dates							
		Mean, (SD)	56.2	(12.0)	56.3	(12.1)	0.755
Sex							
		Female	7373	(54.0)	7385	(54.1)	0.894
		Male	6279	(46.0)	6267	(45.9)	
Comorbidities							
	Hypertension						0.808
		Yes	2689	(19.7)	2705	(19.8)	
		No	10,963	(80.3)	10,947	(80.2)	
	Diabetes						0.518
		Yes	1249	(9.1)	1280	(9.4)	
		No	12,403	(90.9)	12,372	(90.6)	
	Arrhythmia						0.292
		Yes	492	(3.6)	525	(3.8)	
		No	13,160	(96.4)	13,127	(96.2)	
	Coronary heart disease						0.983
		Yes	1145	(8.4)	1143	(8.4)	
		No	12,507	(91.6)	12,509	(91.6)	
Exposure to medications							
	Exposed to Aspirin						0.836
		Yes	1290	(9.4)	1300	(9.5)	
		No	12,362	(90.6)	12,352	(90.5)	
	Exposed to anti-coagulants						1.000
		Yes	1181	(8.7)	1181	(8.7)	
		No	12,471	(91.3)	12,471	(91.3)	
	Exposed to lipid-lowering drugs						0.564
		Yes	1288	(9.4)	1316	(9.6)	
		No	12,364	(90.6)	12,336	(90.4)	
	Exposed to Nitrates						0.720
		Yes	803	(5.9)	817	(6.0)	
		No	12,849	(94.1)	12,835	(94.0)	
Propensity score							
		Mean, (SD)	0.05	(0.01)	0.05	(0.01)	1.000

**Table 2:** Incidence rates, crude hazard ratio, adjusted hazard ratio of stroke in the comparison group and concussion group (*n* = 27,304, 1998–2005).

Stroke during Follow-Up	Total Sample	Comparison Group	Concussion Group	(95% CI) Sig. ^1^
Incidence of stroke (per 1000 person-years)	8.07	6.52	9.63	
Number of occurrences	1248	507	741	
Observed person-years	154,657.3	77,725.9	76,931.4	
Crude hazard ratio (95% CI) ^2^		1.00	1.48	(1.32–1.66) ***
Adjusted hazard ratio (95% CI)		1.00	1.65	(1.47–1.85) ***

^1^ Significance: ***, *p* < 0.001; ^2^ CI: Confidence Interval.

**Table 3:** Incidence rates, crude hazard ratio, adjusted hazard ratio of stroke in the comparison group and concussion group (*n* = 27,304, 1998–2005).

		Comparison Group (95% CI)			Concussion Group (95% CI)			*p*-Value	Sig. ^2^
Hemorrhagic stroke ^1^									
	Crude HR	1.00			1.58	(1.24	−2.02)	<0.001	***
	Adjusted HR	1.00			1.73	(1.36	−2.20)	0.002	**
Ischemic stroke ^1^									
	Crude HR	1.00			1.46	(1.29	−1.66)	<0.001	***
	Adjusted HR	1.00			1.62	(1.43	−1.84)	<0.001	***

^1^ The reference category is the comparison group; ^2^ Significance: **, *p* < 0.01; ***, *p* < 0.001.
